# Phytofabricated bimetallic synthesis of silver-copper nanoparticles using *Aerva lanata* extract to evaluate their potential cytotoxic and antimicrobial activities

**DOI:** 10.1038/s41598-024-51647-x

**Published:** 2024-01-13

**Authors:** Gopishankar Thirumoorthy, Balamuralikrishnan Balasubramanian, Jincy A. George, Aatika Nizam, Praveen Nagella, N. Srinatha, Manikantan Pappuswamy, Amer M. Alanazi, Arun Meyyazhagan, Kannan R. R. Rengasamy, Vasantha Veerappa Lakshmaiah

**Affiliations:** 1 Department of Life Sciences, CHRIST (Deemed to be University), Hosur Rd, Bengaluru, Karnataka 560029 India; 2 Department of Food Science and Biotechnology, College of Life Sciences, Sejong University, Seoul, South Korea; 3 Department of Chemistry, CHRIST (Deemed to be University), Hosur Rd, Bengaluru, Karnataka 560029 India; 4 Department of Physics, RV Institute of Technology and Management, Bengaluru, 560 076 India; 5 Pharmaceutical Chemistry Department, College of Pharmacy, King Saud University, 11451 Riyadh, Saudi Arabia; 6 Laboratory of Natural Products and Medicinal Chemistry (LNPMC), Center for Global Health Research, Saveetha Medical College and Hospital, Saveetha Institute of Medical and Technical Sciences (SIMATS), Thandalam, Chennai, 602105 India; 7 Centre of Excellence for Pharmaceutical Sciences, North-West University, Potchefstroom, 2520 South Africa

**Keywords:** Nanobiotechnology, Plant biotechnology, Microbiology, Molecular biology, Nanoscience and technology

## Abstract

In this study, we demonstrate the green synthesis of bimetallic silver-copper nanoparticles (Ag–Cu NPs) using *Aerva lanata* plant extract. These NPs possess diverse biological properties, including in vitro antioxidant, antibiofilm, and cytotoxic activities. The synthesis involves the reduction of silver nitrate and copper oxide salts mediated by the plant extract, resulting in the formation of crystalline Ag–Cu NPs with a face-centered cubic structure. Characterization techniques confirm the presence of functional groups from the plant extract, acting as stabilizing and reducing agents. The synthesized NPs exhibit uniform-sized spherical morphology ranging from 7 to 12 nm. They demonstrate significant antibacterial activity against *Staphylococcus aureus* and *Pseudomonas aeruginosa*, inhibiting extracellular polysaccharide secretion in a dose-dependent manner. The Ag–Cu NPs also exhibit potent cytotoxic activity against cancerous HeLa cell lines, with an inhibitory concentration (IC_50_) of 17.63 µg mL^−1^. Additionally, they demonstrate strong antioxidant potential, including reducing capability and H_2_O_2_ radical scavenging activity, particularly at high concentrations (240 µg mL^−1^). Overall, these results emphasize the potential of *A. lanata* plant metabolite-driven NPs as effective agents against infectious diseases and cancer.

## Introduction

Bacterial biofilms pose a formidable global health challenge, exhibiting resilience against antibiotics, phagocytosis, and environmental stressors, perpetuating chronic infections, particularly by Staphylococcal and Pseudomonas strains^1^. Biofilm formation entails an intricate, multistep process where bacteria adhere to surfaces, leading to heightened antibiotic resistance. The ensuing microbial consortium releases extracellular polysaccharides (EPS), evading immune surveillance and instigating pathogenesis^2–4^. Recent research reveals a profound connection between biofilms and various cancers, going beyond conventional links to colorectal and gastric cancers. In vivo, malignant cells rely on a complex tumor microenvironment (TME) for crucial support, where communication within the TME leads to significant cellular and genetic diversity. This dynamic environment triggers changes in the extracellular matrix (ECM), epithelial-to-mesenchymal transition (EMT), and immune modulation, creating a microenvironment conducive to cancer progression^5^.

Concurrently, cancer cells and biofilms undergo metabolic adaptations, notably the Warburg effect, optimizing nutrient distribution for ATP generation and heightened glutamine levels essential for growth. Biofilms manifest analogous metabolic strategies, efficiently metabolizing glucose for sustained energy in oxygen-limited conditions^6,7^. The coming together of these common metabolic processes presents a crucial challenge with significant implications for treatment approaches. Biofilms impact cancer through various ways, including triggering inflammation, altering immune responses, producing carcinogenic toxins, and changing host metabolism. The developing concept of the tumor microbiome within the tumor microenvironment closely links with cancer progression, highlighting the need to understand bacterial interactions in cancer and uncover the mechanisms of biofilms for improved diagnostic accuracy and innovative therapeutic strategies^8^. Current scientific investigations seek innovative approaches for precise therapeutic agent delivery, enhancing bacterial mortality and impeding cancer cell viability. Employing nanoparticles is a key method to target specific tissues, such as tumor tissues and biofilms, due to their small size and unique properties. This enables direct drug delivery to biofilm-infected tumor sites, overcoming a major challenge in biofilm treatment—limited drug penetration.

Nanotechnology involves engineering atoms and molecules to synthesize nanoparticles (NPs), solid particles typically 1 to 100 nm in diameter and 1 to 1000 nm in length. These NPs, with amorphous or crystalline structures, serve as versatile carriers for liquids or gases, bridging bulk materials and molecular structures^9,10^. The nanoparticle size varies based on synthesis methods: chemical, physical, and biological. It is crucial to note that chemical and physical methods may pose environmental and organismal risks due to potential toxicity. Hence, the adoption of a green synthesis approach, utilizing biological components like plant extracts, bacteria, yeast, algae, and fungi, is increasingly favored. Biological elements, particularly alkaloids and flavonoids, play pivotal roles in reducing NPs during green synthesis, rendering it more environmentally friendly than chemical and physical methods^11,12^.

NPs fall into two main categories: monometallic nanoparticles (MNPs) with a single metal and bimetallic nanoparticles (BNPs) with two different metals. Recent research highlights the superior attributes of BNPs, driven by their increased surface area, making them particularly significant. BNPs leverage the distinct properties of both metals, resulting in unique combined attributes and diverse applications^13–16^. Silver (Ag) and copper (Cu) NPs, synthesized from biological sources like plants, fungi, bacteria, and algae, exhibit antibacterial, anticancer, anti-biofilm, and antioxidant activities. They emerge as promising candidates in various fields, especially biomedicine, demonstrating potential as carriers for diagnostics, hydrophobic medicines, hyperthermia, and cancer therapeutics. The combination of Ag and Cu into bimetallic NPs has piqued interest for its antimicrobial efficacy against various pathogens and preferential toxicity to cancer cells, positioning it as a valuable tool in combatting multidrug resistance and advancing cancer treatment^17–20^.

This study marks the initial step in elucidating a sustainable methodology for synthesizing Ag–Cu bimetallic nanoparticles (NPs) using *A. lanata* plant extract. The NPs undergo comprehensive characterization, encompassing optical, morphological, and elemental analyses. Of specific interest is the assessment of their biological functionalities, namely antibacterial and antioxidant activities, with a targeted focus on elucidating their pronounced antibiofilm and cytotoxic activities.

## Materials and methods

### Chemicals and materials

Silver nitrate and copper sulfate (analytical grade) were obtained from Sigma Aldrich Bangalore, India. All bacteriological media components such as crystal violet, tryptone, yeast extract, glucose, and sodium chloride, and media such as nutrient both were procured from Hi-Media Laboratories Pvt. Ltd., Mumbai, India. The HeLa & HEK293 cell lines utilized in the experiment was acquired from American Type Culture Collection (ATTC, LG Promochem, Barce-lona, Spain).

### Preparation of *Aerva lanata* plant extract

The *Aerva lanata* plant was sourced from the I-AIM herbal garden and nursery in Bengaluru, India, following the relevant guidelines and regulations set by CHRIST (Deemed to be University). To ensure the reproducibility of the study, the voucher representing the specimen has been deposited within the herbarium at the Department of Life Sciences, Christ (Deemed to be University), and is assigned the identification number CULS_AS_001*.* The collected plant material, representing the entire plant, was washed with double distilled deionized water and subsequently shade-dried for a period of 5 days at room temperature. The dried plants were then finely chopped and coarsely powdered. To prepare the extract, 5 g of the whole plant powder was boiled with 100 mL of double distilled deionized water at 70 °C for 20 min. This solution was further filtered using Whatman No.1 filter paper and centrifuged at 6000 *rpm* for 5 min and the supernatant was stored at 4 °C for experimental use.

### Synthesis of bimetallic Ag–Cu NP’s

Silver nitrate and copper sulphate were used as precursor salts for the biosynthesis of bimetallic Ag–Cu NPs. The synthesis process was performed by mixing a stock solution of both the precursor salts (AgNO_3_ and CuSO_4_) in an equal volume of 60 mL with the defined molar concentration of 0.01 M and boiled 10 min at 80 °C. About 30 mL of plant extract was added. With constant mixing on magnetic stirrer at 40 °C for 1 h. Variation in color was observed from green to dark brown and then finally to black precipitate of bimetallic Ag–Cu NPs. These NPs were collected by centrifuging at 10,000 *rpm* and the pellet was washed thoroughly with double-distilled deionized water, followed by the final rinse with ethanol. Nanoparticles were dried in a hot air oven at 80 °C and stored at 4 °C for further use^21^.

### Characterization

**Figure 1:**
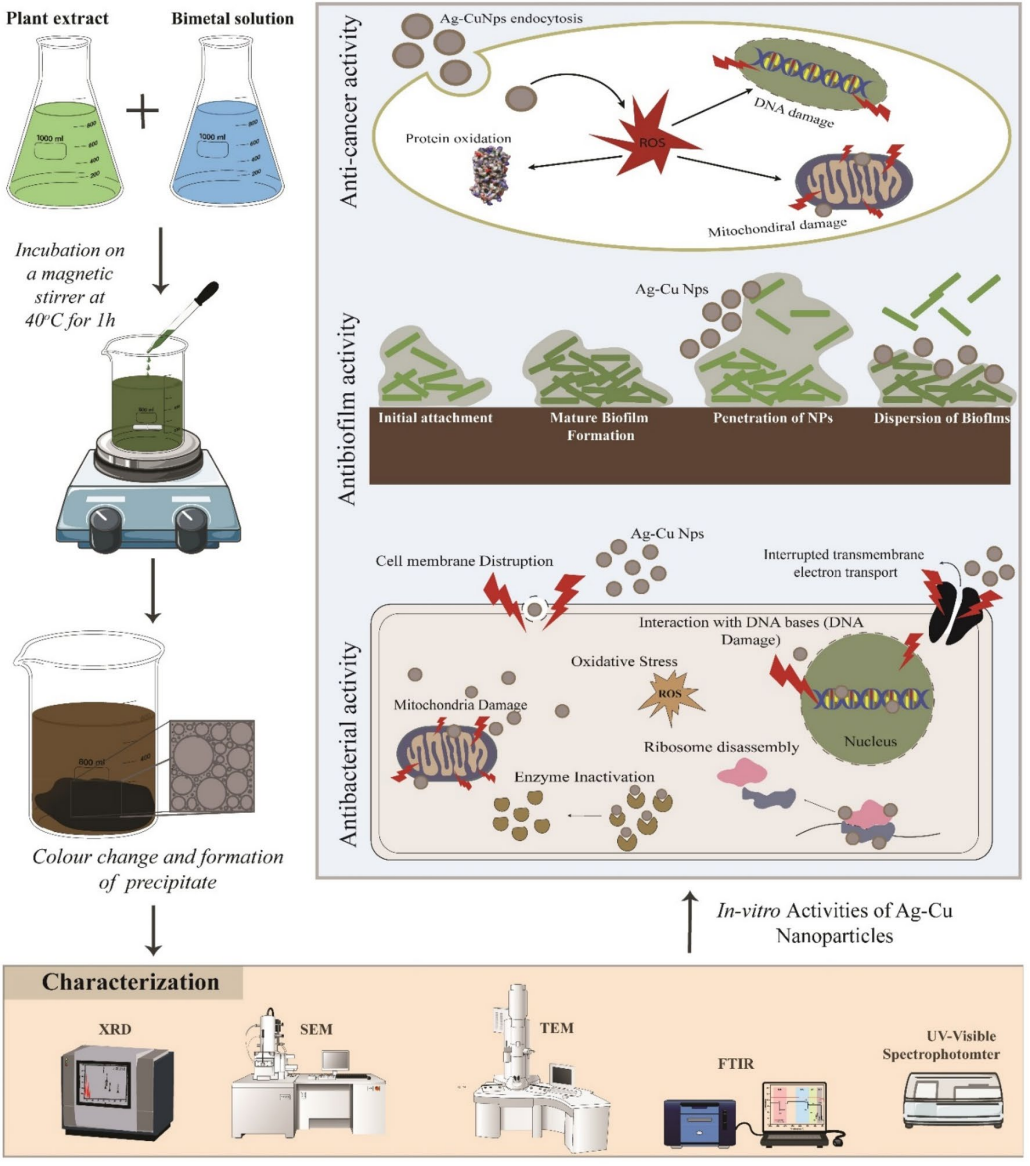
Schematic representation of the study.

Various analytical tools were employed to investigate the key properties of bimetallic NPs synthesized using *A. lanata*. Figure 1 depicts that the schematic study plan and objective. To determine the crystal structure, the Ag–Cu NPs were analyzed using Bragg–Brentano geometry with a fine focus of cu-anode working at 40 kV and 30 mA, utilizing a Shimadzu model XRD 6000 X-ray diffractometer. The spectrum of the bimetallic Ag–Cu NPs was recorded in the 2θ range, with the detector positioned from 10 to 80 degrees in a step scan mode of 0.02°. Fourier-transform infrared spectroscopy (FT-IR) was employed to study the functional groups present in the green-synthesized Ag–Cu NPs. The KBr pellet method was adopted for sample preparation to avoid spectrum adsorption in the IR region and enable the distinction of various functional groups. The FTIR spectrum was measured in the wave number range of 4000–400 cm^–1^. The surface morphology and elemental composition of the synthesized NPs were examined using Field Emission-Scanning Electron Microscope (FE-SEM, model JEOL JSM-6390) and EDX (EDX Oxford Instrument, INCA PentaFETX3). To prevent agglomeration, the Ag–Cu NPs were suspended in a suitable solvent and subjected to sonication. A silicon wafer substrate was then immersed in the colloidal suspension and covered with a spin-coating process, ensuring secure attachment of the particles for SEM and EDX analysis. High Resolution—Transmission Electron Microscope (HR-TEM) analysis was conducted using a Philips TEM (CM200; Eindhoven, The Netherlands) operating at a potential of 120 kV to determine the average particle size and diffraction pattern. The NPs were sonicated and diluted, and the diluted sample was placed on a copper grid and dried at room temperature, HR-TEM images were captured at different magnification powers.

### Determination of minimum inhibitory and minimum bactericidal concentration (MIC and MBC)

The MIC and MBC of the Ag–Cu NPs were tested against *Staphylococcus aureus* and *Pseudomonas aeruginosa*, and results were recorded as per Clinical and Laboratory Standards Institute guidelines. The assay was performed by diluting the concentration of NPs with sterile LB broth to finally obtain series of concentration (15, 30, 60, 120, 240 μg mL^−1^) inoculated with *S. aureus* and *P. aeruginosa* adjusted to the turbidity of 5 × 10^5^ CFU/mL. These incubated broths were incubated on shaker incubator maintained at 37 °C for 24 h. The concentration at which there was no cell density or turbidity was recorded as MIC^22^. Following the Ag–Cu NPs MIC measurement, 30 μL aliquots from the tubes that were plated on MH agar plates and incubated overnight at 37 °C overnight. The lowest concentration of NPs that reported bacterial killing was reported to be the MBC value.

### Well diffusion assay

Agar well diffusion technique was adopted to study the anti-microbial activity of the Ag–Cu NPs. This assay was performed with two bacterial species *S. aureus* and *P. aeruginosa*. A revived mid log phase bacterial cultures were spread plated on Muller Hinton agar. Followed by punching of the wells in the solidified media using gel puncher that resulted in wells with a diameter of 5 mm. Under aseptic conditions the wells were loaded with different concentrations of Ag–Cu NPs from 15, 30, 60, 120 μg mL^−1^ and positive control ampicillin. The sample loaded culture plate was then incubated at 37 °C for overnight to observe and record zone of clearance in the plates and the diameter of the zones was measured^23^.

### Effect of Ag–Cu NPs on biofilm formation

#### Biofilm formation assay

The test organism in LB broth was inoculated and allowed to grow until reaching a constant turbidity of 0.5 McFarland standards (5 × 10^5^ CFU/mL). Different concentrations (15, 30, 60, 120, 240 μg mL^−1^) of Ag–Cu NPs were added to separate tubes containing the bacterial suspension and incubated at 37 °C for 24 h. A negative control without the addition of NPs was also maintained. After incubation, the media was removed, and the tubes were washed three times with phosphate buffer saline (PBS, pH 7.2). Subsequently, the tubes were stained with 0.1% crystal violet dye for 30 min. Excess stain was washed off with deionized water, and the tubes were dried at room temperature. The biofilm formation was assessed by observing the presence of a thin blue film on the walls of the tubes^24^.

To quantitatively estimate the effectiveness of Ag–Cu NPs in inhibiting biofilm formation, a method proposed by Kalishwaralal et al. was employed using a 96-well microtiter plate^25^. Briefly, 10 μL of overnight grown and diluted (1:100) culture was added to sterile wells containing 180 μL of LB broth. Ag–Cu NPs were then added at concentrations of 15, 30, 60, 120, 240 μg mL^−1^. After incubation at 37 °C for 24 h, the LB broth was discarded, and the wells were washed three times with phosphate buffer saline (PBS, pH 7.2) to remove any unbound cells. The biofilm adhered to the well walls was fixed with 2% w/v sodium acetate and stained with 0.1% crystal violet dye for 10 min. The stained cells were washed with sterile double distilled deionized water, and the crystal violet stain on the surface of the biofilm was solubilized by adding 30% acetic acid and incubating at room temperature for 10–15 min. The solubilized crystal violet was transferred to a new 96-well plate, and the absorbance at 570 nm (OD 570) was measured using an ELISA plate reader. The percentage of biofilm inhibition was calculated using the provided formula:

1 $$\text{Inhibition (\%) = }\frac{\left(\left({\text{OD}}_{\text{control}}\text{ - }{\text{OD}}_{\text{treated}}\right)\right)}{{\text{OD}}_{\text{control}}} \, \times \text{100 .}$$

### Effect of Ag–Cu NPs on the production of extracellular polymeric substance (EPS)

The inhibitory influence of Ag–Cu NPs on the production of EPS was quantitatively analyzed slightly modified assay^26^. An overnight culture of *P. aeruginosa* and *S. aureus* aseptically inoculated in 50 mL sterile LB broth and each of these cultures were subjected to different sub-inhibitory MIC concentrations of NPs (15, 30, 60, 120, 240 μg mL^−1^) and one without NPs was maintained as negative control. All the tubes were incubated at 37 °C for 24 h in a shaker incubator at 140 *rpm*. After incubation period, the control and the treated samples were centrifuged at 6000 *rpm* for 15 min at 4 °C and the supernatant was collected. Supernatant obtained was treated with double the volumes of ice-cold ethanol followed by refrigeration at 4 °C to facilitate the precipitation of EPS. This precipitation was separated and concentrated by centrifugation at 6000 *rpm* for 20 min at 4 °C. The obtained pellet was used for the quantification by measuring carbohydrates utilizing anthrone method^27^. The percentage of EPS inhibition was calculated using Eq. (1).

### Analysis of antioxidant activity of the synthesized bimetallic Ag–Cu NPs

#### Reducing power assay

The reducing power of the NPs was determined according to the method of Alavi and Karimi with some modifications^28^. The concentrations of the bimetallic NPs from 15, 30, 60, 120, 240 μg mL^−1^ were prepared using 0.2 M of phosphate buffer of pH 6.4. The aliquots were mixed with 2.5 mL of 10 mL^–1^ concentration of potassium ferricyanide and incubated at 50 °C for 30 min. After the incubation, 2.5 mL of 100 mL^-1^ concentration of trichloro acetic acid was added and centrifuged at 10,000 *rpm* for 12 min. The supernatant was mixed with equal volume of distilled water (3 mL), 0.6 mL of 1.0 mL concentration of ferric chloride was added. With BHT as control setup, the solution was read for absorbance at 700 nm. The results indicate that more the absorbance, the higher is its reducing power.

#### Hydrogen peroxide scavenging assay

The hydrogen peroxide scavenging assay was performed by modifying the methods of Vilas et al.^29^, with ascorbic acid as control, various concentration of the bimetallic NPs (15, 30, 60, 120, 240 μg mL^−1^) was aliquoted and added with 100 μL of 3 mM hydrogen peroxide solution. The solution was incubated at room temperature for 30 min and the absorbance was measured at 610 nm. The percentage of scavenging was calculated using Eq. (1).

#### Cytotoxicity assay

##### MTT assay for testing cell viability

The effect of biogenic Ag–Cu NPs on HeLa and HEK293 cell lines was investigated using the MTT assay (3-(4,5-dimethylthiazol-2-yl)-2,5-diphenyltetrazolium bromide) at various concentrations. Briefly, the metabolic rate and viability of the treated cells were assessed by measuring the reduction of the yellow tetrazolium salt MTT to a blue formazan product by mitochondrial dehydrogenase. The cells were seeded in a 96-well plate at a uniform density of 1 × 10^4^ cells/well and allowed to adhere and proliferate overnight in a 5% CO_2_ incubator. After a 24 h incubation period, the monolayer was washed with fresh medium, and the adhered cells were exposed to Ag–Cu NPs at concentrations ranging from 0 to 240 µg mL^–1^ for an additional 24 h. To monitor cell viability, 10 μL of MTT prepared with PBS was added to each well. The plates were then incubated in darkness at 37 °C in a humidified atmosphere containing 5% CO_2_ for 4 h. The supernatant was mixed with 100 μL of DMSO to solubilize the formazan product. The absorbance of the plates was measured at 550 nm^30^. A blank well containing medium without bimetallic nanoparticles at the corresponding concentrations was included. The percentage of inhibition was calculated using a specific formula, and the concentration of nanoparticles required to inhibit cell growth by 50% (IC_50_) values was determined from the dose–response curves for each cell line using GraphPad Prism 7.0.

### Statistical analysis

The experiments were conducted in triplicate and analyzed using Student’s *t* test and two-way ANOVA, with statistical significance defined as p-values < 0.01, utilizing GraphPad Prism software (GraphPad Software Tools, Inc., La Jolla, CA, USA).

## Results and discussions

### Synthesis of Ag–Cu NPs

The plant chosen for this research belongs to the *Amaranthaceae* family and is found extensively in tropical and subtropical regions around the world. Due to its rich composition of phytochemicals, including alkaloids, terpenoids, sterols, flavonoid glycosides, and polyphenols, it holds significant importance in ayurveda and is utilized for various medicinal purposes such as antimicrobial, antiasthmatic, anti-urolithiasis and anti-hyperglycemic^31,32^. In recent studies, numerous investigations have confirmed the viability of utilizing *A. lanata* plant extract for the synthesis of different metal nanoparticles. The presence of phytochemicals in the plant extract acts as an essential reducing or capping agent, facilitating the synthesis of metallic nanoparticles^33–36^. Upon combining the plant extract with the precursor salt, the color of the solution undergoes a discernible change from green to black after an hour of incubation. This color transformation serves as confirmation of the reduction process facilitated by the plant's metabolites. Eventually, the resulting precipitate settles down undisturbed, indicating the successful reduction and formation of bimetallic Ag–Cu NPs.

### Characterization of bimetallic Ag–Cu NPs

#### X-ray diffraction

**Figure 2:**
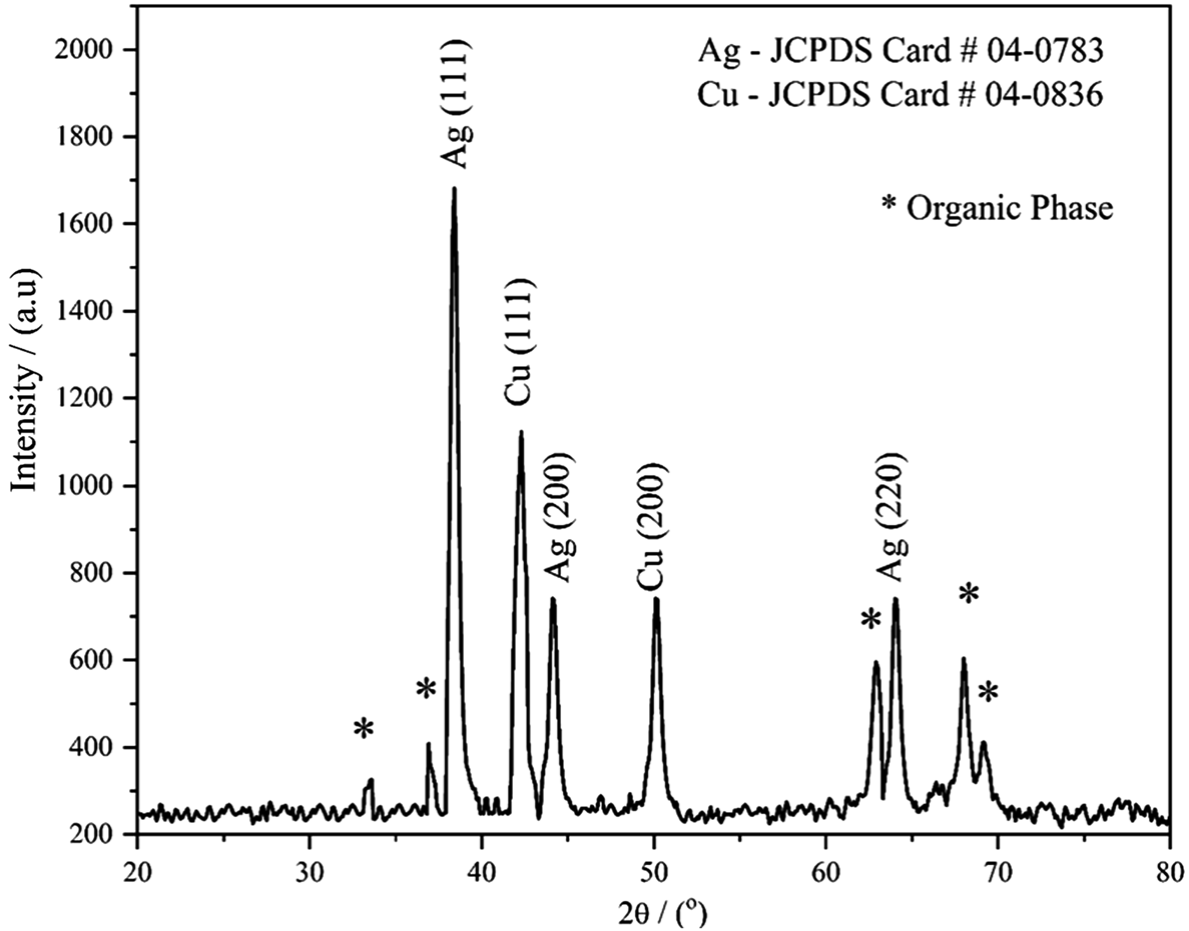
The XRD pattern of green synthesized bimetallic Ag–Cu nanoparticles.

The phase—purity and the crystallinity of the prepared bimetallic Ag–Cu NPs were studied using XRD technique. The XRD pattern of the prepared sample is depicted in Fig. 2. It is observed that, XRD pattern exhibits sharp, well defined high peaks. In detail, the peaks observed at 38.3°, 44.1° and 64.1° in 2θ are assigned to the (111), (200) and (220) planes which corresponds to the JCPDS card no. 04 - 0783 belongs to the face centered cubic (fcc) crystal structures of Ag^37^. The crystalline peaks observed at 43.0° and 50.1° are attributed to the (111) and (200) planes of fcc cubic structured Cu NPs corresponds to JCPDS card no. 04 - 0836^38^. In addition to the Ag and Cu crystalline peaks, certain unassigned peaks (marks with *) are also observed. The existence of these peaks could be due to the crystalline organic matter induced during the synthesis via plant extract. These findings show that Cu–Ag NPs produced using *A. lanata* extract results in the nano crystalline particles. The average crystallite size was estimated using Debye – Scherrer equation; D = kλ/βcosθ; where β is the full width at half maximum and λ is the x-ray wavelength (1.5418 Å) and k is the constant. All the peaks obtained in the study were used to estimate the size crystallites and the average size of the crystallite found be 14.6 nm that is in correspondence to previously reported Cu–Ag NPs^39^.

#### FT-IR

**Figure 3:**
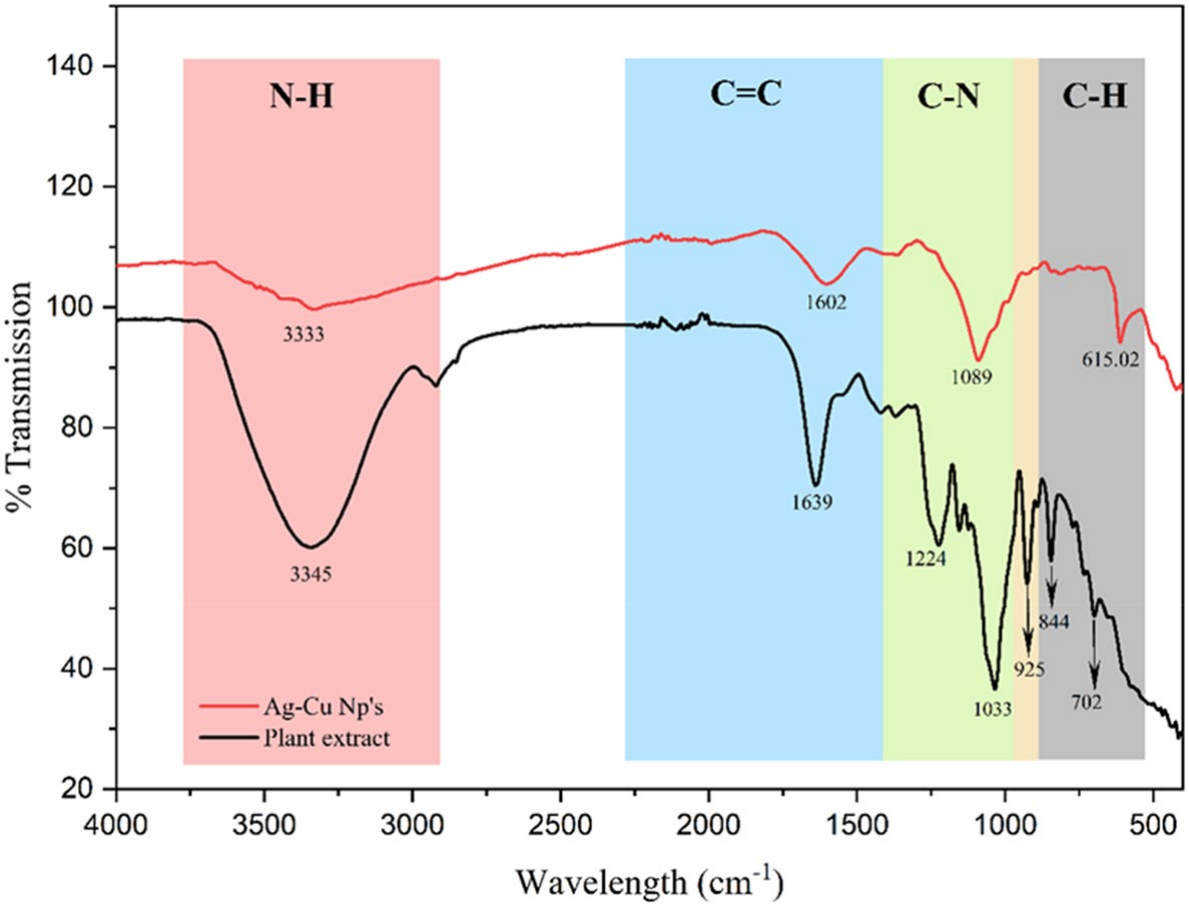
The FTIR analysis spectra of *Aerva lanata* extract and green synthesized Ag–Cu NPs.

The FT-IR study was conducted to analyze the functional group of the tethered capping biomolecule that aided in the synthesis and stabilization of the bimetallic Ag–Cu NPs. Both the extract and the NPs were subjected to IR radiation within the range from 400 to 4000 cm^–1^. FT-IR spectrum of *A. lanata* extract exhibited absorption bands at 3345, 1639, 1224, 1033, 925, 844, 702 cm^–1^ which corresponds to stretching vibration of medium N–H stretching (aliphatic primary amine), bending of –OH, C=O stretching of strong alkyl aryl ether, C–N variable stretching, O–H stretching and N–H wag^39^. For the spectrum of Ag–Cu/Al, the characteristic peaks of the *A. lanata* extract were found together with a new medium peak of 615 cm^–1^ correlating the C–H bond indicating nanoparticles of Ag–Cu and validating that Ag–Cu NPs were capped with phytoconstituents of *A. lanata*. Furthermore, when the *A. lanata* extract and Ag–Cu NPs were compared (Fig. 3), the peaks of hydroxyl, carbonyl, and amino groups were found to slightly shifted in the NPs, confirming the interaction and participation of bioactive molecules in the production of Ag–Cu NPs. These findings corroborated the utilization of bioactive *A. lanata* components on the surface of Ag–Cu NPs as capping, reducing, and stabilizing agents^11,40^.

### Field emission—scanning electron microscope (FE-SEM) and EDX

**Figure 4:**
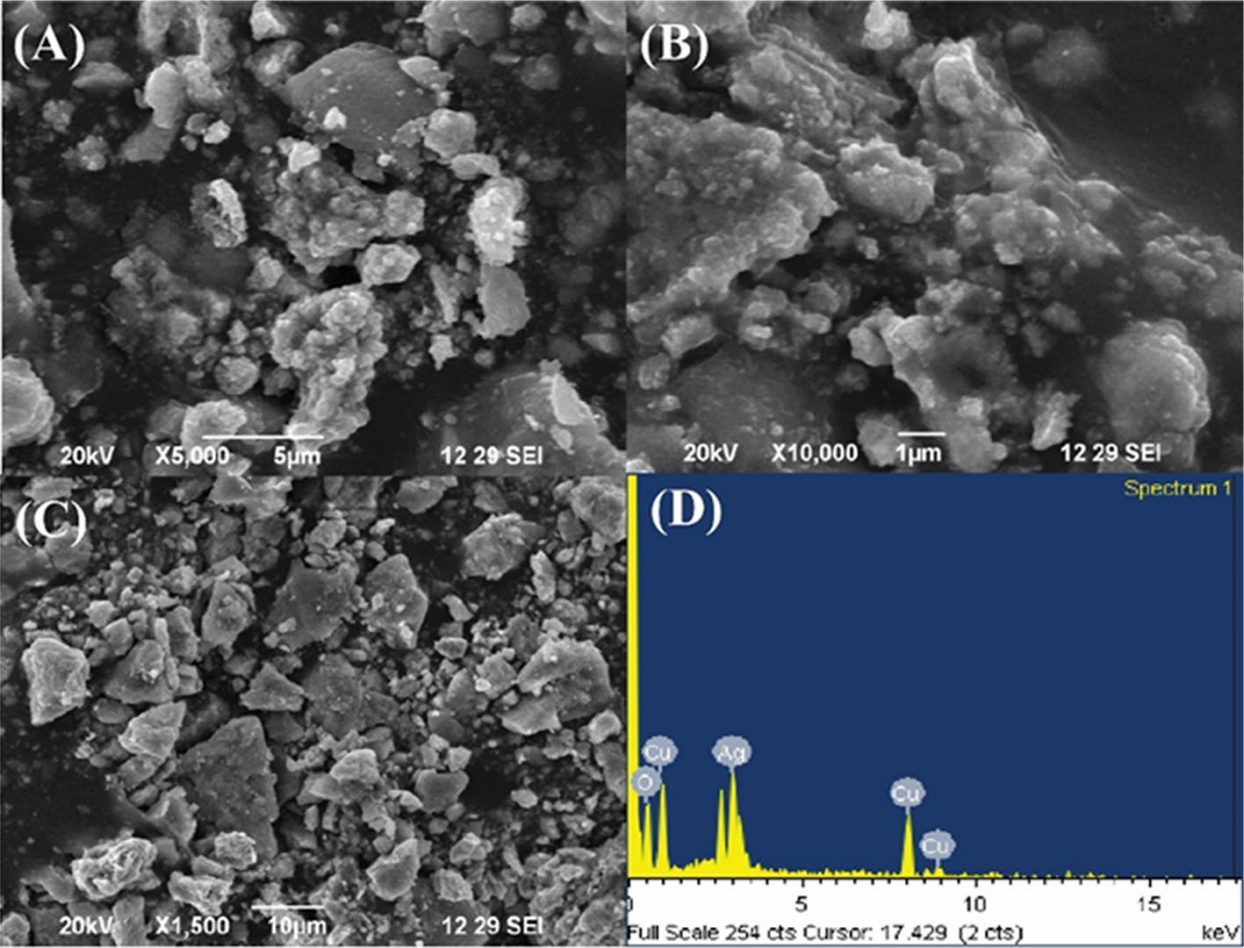
(**A**–**C**) SEM images at different magnifications and (**D**) EDAX spectra of green synthesized bimetallic Ag–Cu NPs.

The SEM images, presented in Fig. 4, depict the bimetallic Ag–Cu NPs that were synthesized using a green method. The images reveal that these NPs have a varied size distribution and form semi-spherical agglomerated clusters with a combination of different shapes. Additionally, a significant portion of the NPs exhibit rough surfaces. The EDAX spectra were used to perform a semi-quantitative elemental mapping of the bimetallic Ag–Cu NPs. The results from EDAX confirm the presence of both Ag and Cu particles, indicating their bimetallic nature^41^.

### High resolution—transmission electron microscope (HR-TEM)

**Figure 5:**
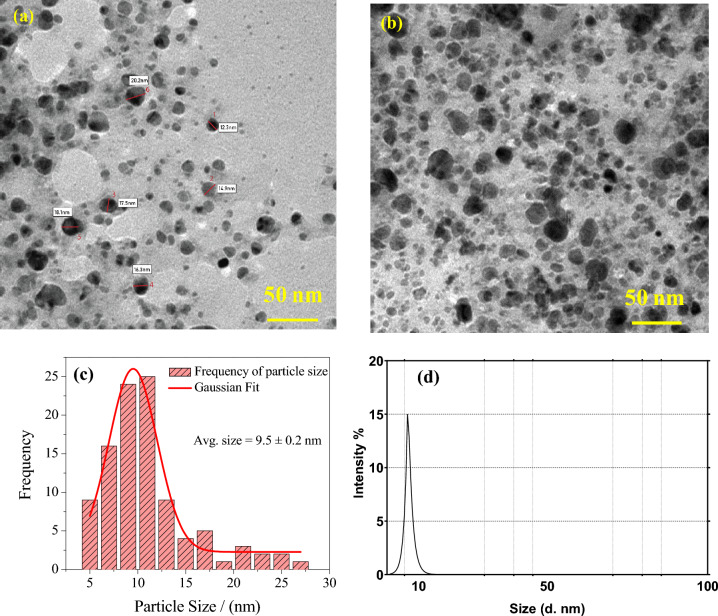
(**a**) and (**b**) HR-TEM images of Ag–Cu NPs; (**c**) showing the histogram with the Gaussian fitted particle size distribution; (**d**) DLS graph exhibiting the hydrodynamic diameter of the nanoparticles.

The HR-TEM technique was employed to analyze the size distribution and morphology of the bimetallic NPs. The HR-TEM images, along with the corresponding size distribution histogram, are presented in Fig. 5. Upon examining the HR-TEM images (Fig. 5a,b), it can be observed that the particles are uniformly dispersed and exhibit a wide size distribution. The particle sizes were measured using Image J software, and the corresponding size distribution histogram is shown in Fig. 5c. The histogram reveals that the particle sizes range from 5 to 30 nm, with the majority falling within the 7 nm to 12 nm range, this was further confirmed by dynamic light scattering analysis Fig. 5d. The average size of the particles is approximately 9.5 nm, which aligns well with the crystallite size estimated from XRD analysis. It is worth noting that the bimetallic Ag–Cu NPs synthesized through the green method exhibit a spherical shape. The presence of larger particles may be attributed to the coagulation or overlapping of smaller particles, a phenomenon that has been reported in similar studies conducted by Al Tamimi, S. et al.^42^.

### Determination of minimum inhibitory concentration

The MIC and MBC of the biosynthesized NPs against the desired organisms was evaluated. It was shown that the MIC and MBC of Ag–Cu NPs against *P. aeruginosa* and *S. aureus* was 240 and 120 μg mL^−1^ respectively. The findings demonstrate that the effectiveness of Ag–Cu NPs was more potent for *S. aureus* than *P. aeruginosa*. This is due to the presence of positive charged silver in the bimetallic NPs that trap and block the lipopolysaccharides core component of bacterial cell wall of Gram-negative bacteria *P. aeruginosa*, finally them susceptible for bimetallic NPs. Along with this morphology and surface-to-mass ratio of NPs influence their reactivity against microbes^43^. It was also implied that, due to its biological and chemical reactive edges, uneven and irregular particles have various binding features with the microbial surface. The plant-based synthesis resulted in a wide range of particle sizes. The findings of this study are in moderate correlation with NPs XRD results. A prominent peak of 111 facets can be seen in the bimetallic NPs. The XRD pattern is closely related to TEM analysis, emphasizing that that majority of synthesized NPs are spherical shaped nanoparticles in the sample with high 111 facets, which enhanced their antibacterial activity^44^.

### Agar well diffusion method

**Figure 6:**
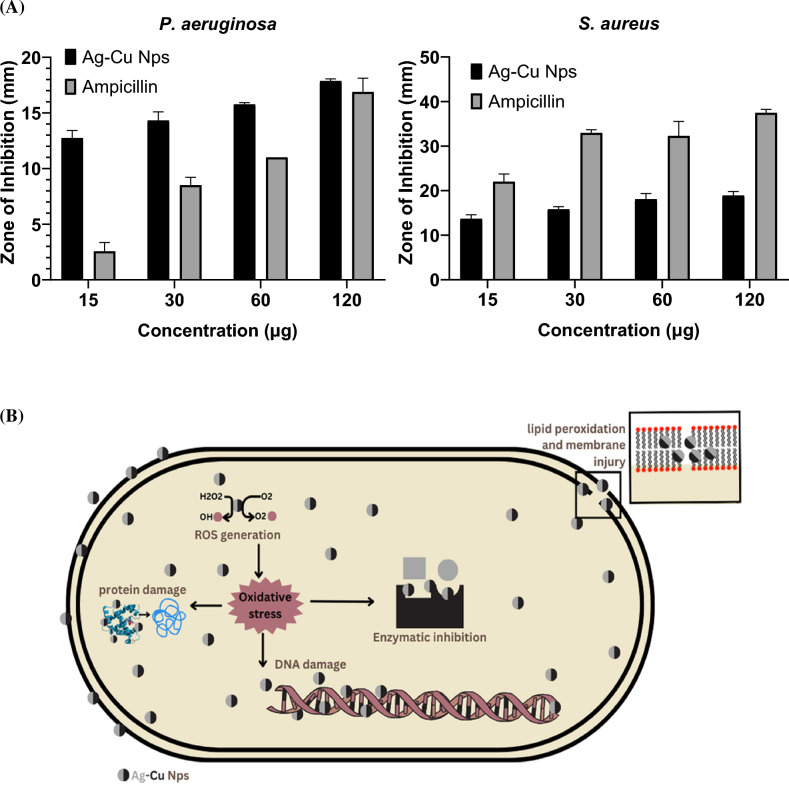
(**A**) Inhibition zones observed for Ag–Cu NPs and Ampicillin (at concentrations ranging from 15 to 120 µg mL^–1^) against *P. aeruginosa* and *S. aureus*. (**B**) Mechanism of action underlying the antibacterial activity of Ag–Cu nanoparticles (NPs).

The anti-bacterial activity of the green synthesized Ag–Cu NPs was qualitatively evaluated using well diffusion method against Gram positive (*S. aureus*) and Gram negative (*P. aeruginosa*) organisms. The results of the antibacterial activity are presented in Fig. 6A, Table 1 and Figs. S1, S2. Figure 6A illustrates that the highest inhibitory activity of the Ag–Cu NPs was observed at a concentration of 120 μg mL^–1^ against both the organisms. When compared to the control antibiotic, Ampicillin, the activity of the NPs against *P. aeruginosa* was found to be more effective, while against *S. aureus*, it was recorded as moderate. In comparison to the antibacterial activity of the plant extract as reported by Al-Ansari et al., as well as the monometallic Ag and Cu NPs reported by Naidu et al. and Thanganadar Appapalam & Panchamoorthy et al. our bimetallic nanoparticles exhibited superior efficacy^45–47^. They demonstrated significantly larger zones of inhibition at lower concentrations. This enhanced activity attributed to its unique property of large surface area ratio (less particle size) of the NPs which enables accumulation and penetration of the NPs through the external barrier cell wall followed by the generation of reactive oxygen species (ROS) a stress marker which could damage the cell integrity by the disruption of the cell membrane leading to the leakage of membrane proteins and cell lysis^48^ (Fig. 6B). According to Slavin et al. the copper ions have more affinity towards the gram-positive organisms mainly because of projected functional groups such as carboxyl and amine functional groups on the cell surface^49^.

**Table 1:** Summary of the anti-bacterial activity of Ag–Cu NPs.

Organisms	Zone of inhibition (mm)			
	Different concentrations			
	15 μg mL^−1^	30 μg mL^−1^	60 μg mL^−1^	120 μg mL^−1^
*Staphylococcus aureus*	13.0 ± 0.3	15.3 ± 0.4	17.1 ± 0.6	18.0 ± 0.2
*Pseudomonas aeruginosa*	12.2 ± 0.4	14.0 ± 0.7	15.6 ± 0.5	17.7 ± 0.2

### Crystal violet blue assay

**Figure 7:**
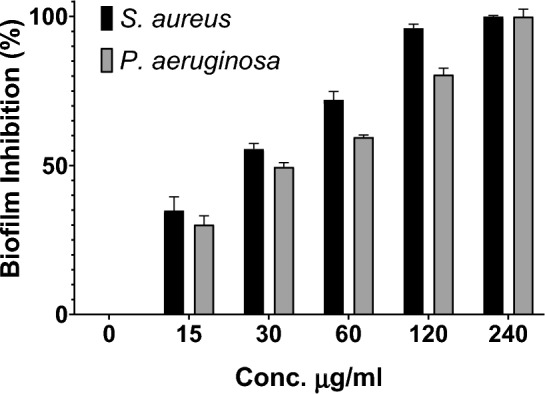
Effect of Ag–Cu NPs on biofilm production by *S. aureus* and *P. aeruginosa*.

The objective of this assay was to investigate the impact of Ag–Cu nanoparticles (NPs) on biofilm formation, both quantitatively and qualitatively, using *S. aureus* and *P. aeruginosa* as test microbes. Sub-inhibitory concentrations of Ag–Cu NPs were used, and the results demon-started their inhibitory effect on biofilm formation. A comparative study was conducted, showing that the presence of bimetallic NPs inhibited biofilm formation without affecting the control group, and this effect was concentration-dependent. Qualitative evaluation involved observing the presence of a thin layer of biofilms on the walls of the culture tubes after staining with crystal violet. The findings indicated that *S. aureus* did not exhibit visible biofilm formation in the culture tubes containing Ag–Cu NPs concentrations of 120 μg/mL and higher. Similarly, *P. aeruginosa* showed no visible biofilm formation at concentrations of 240 μg/mL (Table 2). The quantitative assessment of biofilm inhibition was conducted using a microtiter plate assay at concentrations ranging from 15 to 240 μg/mL, with the inhibition percentage re-ported in Fig. 7. These results align with a study conducted by Ghosh, S. et al. which examined the effect of AucoreAgshell NPs synthesized using *Dioscorea bulbifera* plant extract on biofilm production by *P. aeruginosa*^50^. The Au_core_Ag_shell_ NPs showed an efficiency of 18.93% inhibition of biofilms at a concentration of 100 μg/mL.

**Table 2:** Percentage (%) inhibition of biofilm by Ag–Cu NPs.

Organisms	15 μg mL^−1^	30 μg mL^−1^	60 μg mL^−1^	120 μg mL^−1^	240 μg mL^−1^
*Staphylococcus aureus*	25.09	40.08	71.72	97.43	100
*Pseudomonas aeruginosa*	20.52	37.65	68.71	85.55	100

### Effect of Ag–Cu NPs on the production of extracellular polymeric substance (EPS)

**Figure 8:**
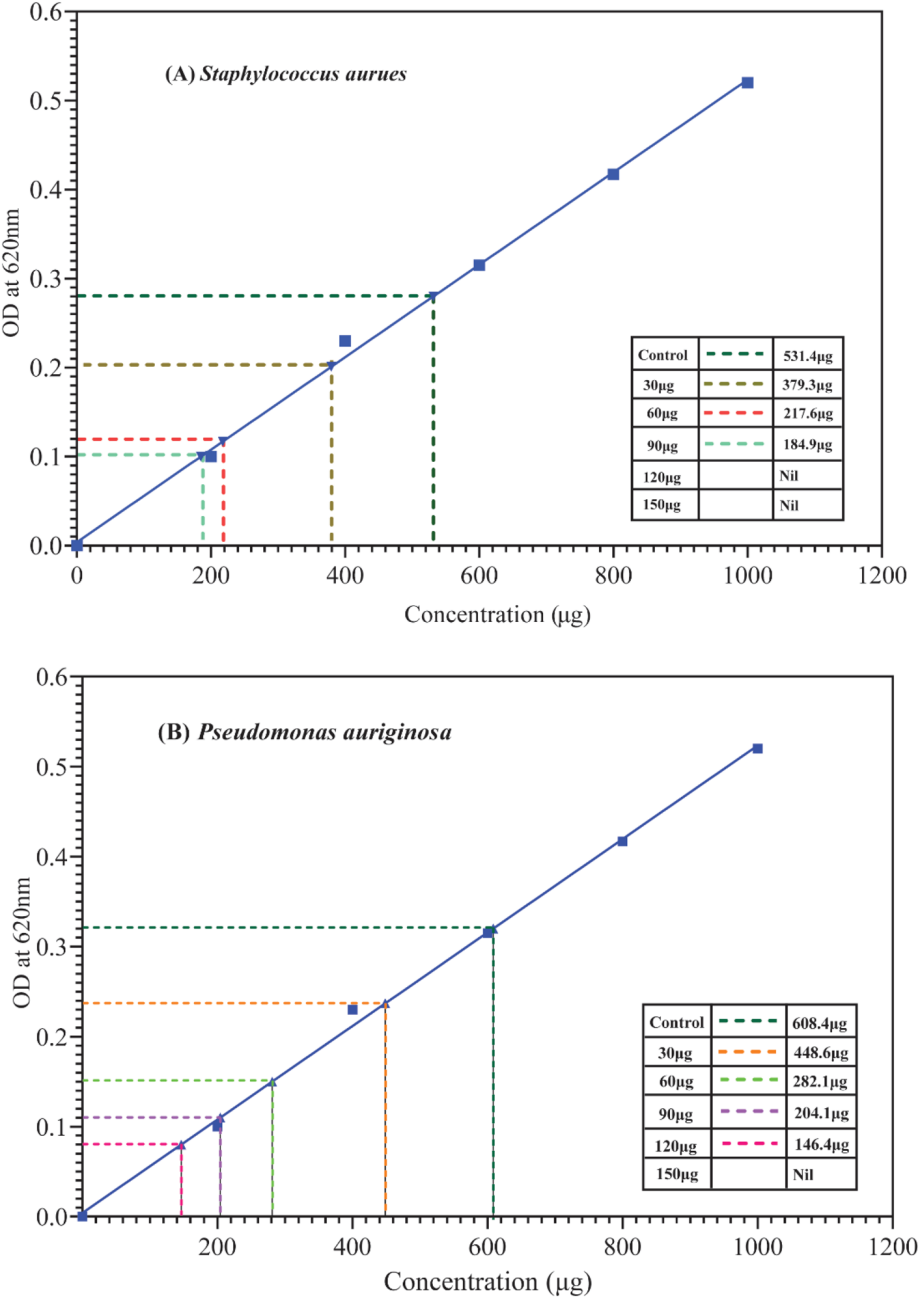
In comparison with the control the inhibition of carbohydrates in EPS produced from *S. aureus* (**A**) was found to be at the concentration of 120 and 150 μg mL^−1^and for *P. aeruginosa* (**B**) it was found to be at the concentration of 150 μg mL^−1^, followed by a gradual decrease in both the bacterial strains.

Carbohydrates are considered as the major constituents for EPS in a pure culture that can mediate the process of biofilm formation. Current study data obtained clearly suggests that the bimetallic Ag–Cu NPs have potent antibiofilm activity by disrupting the EPS matrix. These Ag–Cu NPs exhibited potent EPS degradation against both the bacterial strains in a dose de-pendent manner (15, 30, 60, 120, and 240 μg mL^–1^). The qualitative observation was done by observing the formation of precipitate after the incubation time of 48 h. At a higher concentration of 120 and 240 μg mL^−1^ Ag–CuNPs had manifested a prominent inhibitory effect on the EPS formation with *S. aureus* moderate effect observed with *P. aeruginosa* however the highest concentration of 240 μg mL^−1^ manifested the disruption of EPS against *P. aeruginosa* as well. Anthrone method was found to be a reliable and highly sensitive method to estimate EPS (carbohydrate) and to interpret the inhibitory effect of Ag–Cu NPs (Figs. 8a,b). The ratio of exopolysaccharide was comparatively less after the exposure of the bacterial cultures with the sub-inhibitory concentrations of NPs. From Table 3 it was shown that the highest percentage reduction of EPS was observed against *S. aureus* and *P. aeruginosa* with respect to the concentration of 120 and 240 μg mL^−1^ which is considered to be crucial for biofilm formation. In contrast to our findings, Borcherding et al. reported that when *P. aeruginosa* culture was treated with super paramagnetic iron oxide nanoparticles it demonstrated a significant increase in biofilm biomass^51^. This was attributed to the nutritional role of the iron NPs which supports microbial growth leading to the development of increased microbial biofilm. This demands extensive research to understand the differential effects of various NPs towards cell density and biofilm.

**Table 3:** Comparison of carbohydrate concentration and carbohydrate inhibition & of the samples with control.

Organisms	Carbohydrate concentration (µg) & Carbohydrate Inhibition (%) in control and treated samples										
	C	%	15 μg mL^−1^	%	30 μg mL^−1^	%	60 μg mL^−1^	%	120 μg mL^−1^	%	240 μg mL^−1^
*P. aeruginosa*	608.4	–	448.6	25.43	282.1	53.12	204.1	64.62	146.4	75.00	–
*S. aureus*	531.4	–	379.3	28.21	217.6	58.21	184.9	68.28	–	–	–

*C* control.

### Assessment of Ag–Cu Nps cytotoxic activity

**Figure 9:**
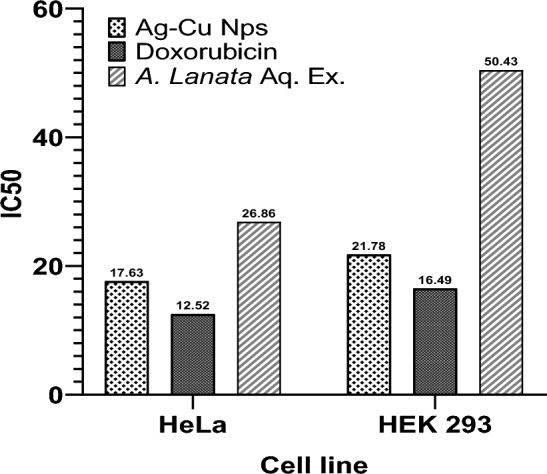
IC_50_ of *A. Lanata* aqueous extract, Ag–Cu NPs and Doxorubicin on the HeLa cancer cells compared to HEK293 normal cells.

The cytotoxicity of *A. Lanata* aqueous extract and Ag–Cu NPs was investigated on HeLa and HEK293 cell lines, with doxorubicin serving as the positive control, and untreated cells as the negative control, using the MTT assay. The resulting MTT assay results are graphically depicted in Fig. 9. Markedly, the IC50 values of HeLa cells were 15.86 μg mL^−1^for the extract and 17.63 μg mL^−1^ for Ag–Cu NPs, respectively. In stark contrast, significantly higher IC50 values of 35.89 μg mL^−1^ and 21.78 μg mL^−1^ were obtained from the treatment of normal human embryonic kidney (HEK293) cells with the respective materials. Meanwhile, similar in range IC50 values were observed among the different cell lines treated with Doxorubicin, with values of 12.52 and 16.490 μM mL^−1^ for HeLa and HEK293 cell lines, respectively. This is consistent with the microscopic examination data, through which the cytotoxic activity could be distinguished by features such as membrane blebbing, cell swelling, or shrinkage, as shown in Figs. S3, S4. This study strongly suggests that the synergistic effect arising from the combined presence of silver and copper in the nanoparticles significantly contributes to their efficacy. When comparing the response of HeLa and normal HEK 293 cells, it was observed that the bimetallic nature of the nanoparticles exhibited lower toxicity towards non-cancerous cells, particularly at a concentration of 120 μg mL^−1^. This phenomenon can be attributed to the antioxidant properties of the NPs, resulting in higher toxicity towards cancerous cells and lower toxicity towards healthy cells, possibly through the expression of apoptotic molecules^52,53^. Further comprehensive investigations are warranted to gain a deeper understanding of the underlying mechanisms responsible for the anticancer activity of the synthesized bimetal NPs.

### Antioxidant activity

**Figure 10:**
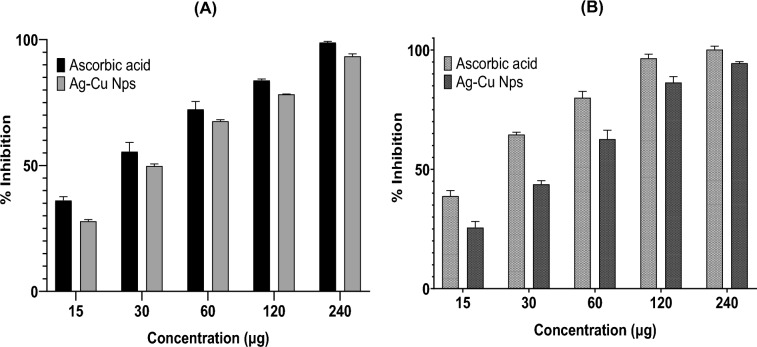
The highest OH– free radical scavenging activity was observed in the concentration of 240 μg mL^−1^ in both Hydrogen peroxide scavenging assay (**A**) and reducing power (**B**); however, the activity was less when compared with the standard AA.

Antioxidant activity is a complex process influenced by various mechanisms and factors, making it challenging to fully comprehend using a single methodology. To capture the diverse mechanisms of antioxidant action, it is necessary to employ multiple evaluation methods for assessing antioxidant capacity. In this study, two complementary tests, namely the reducing power assay and the hydroxyl radical scavenging assay, were utilized to evaluate the anti-oxidant activity of bimetallic Ag–Cu NPs. The reducing power assay measures antioxidant activity by reducing ferric cyanide complex (Fe^3+^) to the ferrocyanide form (Fe^2+^). The presence of antioxidative properties in the NPs enables them to donate an electron, resulting in a color change from green to blue, which indicates their scavenging ability^29^. The results of this study revealed that the NPs exhibited a reduction ability, as evidenced by the color change to Perl's Prussian blue observed at 700 nm. The scavenging activity was assessed in comparison to ascorbic acid, a well-known standard antioxidant. Notably, the nanoparticles exhibited the most substantial reduction in Fig. 10A and B when employed at a concentration of 240 µg mL^−1^. Importantly, it is worth highlighting that, in accordance with Al-Ansari et al. research, the plant extract exhibited 58.5% antioxidant activity at its highest concentration (100 µg), whereas the synthesized Ag–Cu nanoparticles achieved ~ 60 ± 5% of activity at a lower concentration (60 µg mL^−1^)^46^. These findings indicates that the nanoparticles are capable of converting the ferric cyanide complex (Fe^3+^) into the ferrocyanide form (Fe^2+^) and effectively neutralizing hydroxyl (OH–) free radicals^54^. Moreover, the effectiveness of this scavenging activity is influenced by the dosage of the nanoparticles administered.

## Conclusion

The present study successfully demonstrates the development of an eco-friendly, rapid, and cost-efficient method for synthesizing Ag–Cu NPs using *Aerva lanata* extract, fabricated through a phytofabrication process, exhibit great potential for applications in therapeutics as antibiofilm treatments. The FT-IR analysis indicates the presence of hydroxyl, carbonyl, and amino groups in the *A. lanata* extract, which play a significant role as capping agents for the Ag–Cu NPs. The green synthesis of Ag–Cu NPs resulted in a face-centered cubic crystal structure with an average particle size of 9.5 nm. Moreover, these nanoparticles exhibit promising activities in terms of cytotoxicity, antibacterial, and antioxidant properties, attributed to the intrinsic hydroxy and phenol capping, thus suggesting their potential for broader applications. These bimetallic nanoparticles hold promise for applications in combating bio-films. which are crucial factors in various fields such as healthcare and industry. Additionally, the Ag–Cu NPs demonstrate remarkable anti-cancer, antibacterial, and antioxidant activities, making them potential candidates for further research and development in the pursuit of novel therapeutic interventions.

### Supplementary Information

Supplementary Figures.

## Data Availability

The data presented in this study are available on request from the corresponding authors.
