# Supplementary material for: Phytofabricated bimetallic synthesis of silver-copper nanoparticles using Aerva lanata extract to evaluate their potential cytotoxic and antimicrobial activities
Source: Sci Rep. 2024 Jan 13;14:1270. doi: 10.1038/s41598-024-51647-x (PMC10787839; doi:10.1038/s41598-024-51647-x)
Supplement: Supplementary file 1 — Supplementary Figures. [file 41598_2024_51647_MOESM1_ESM.docx]

**
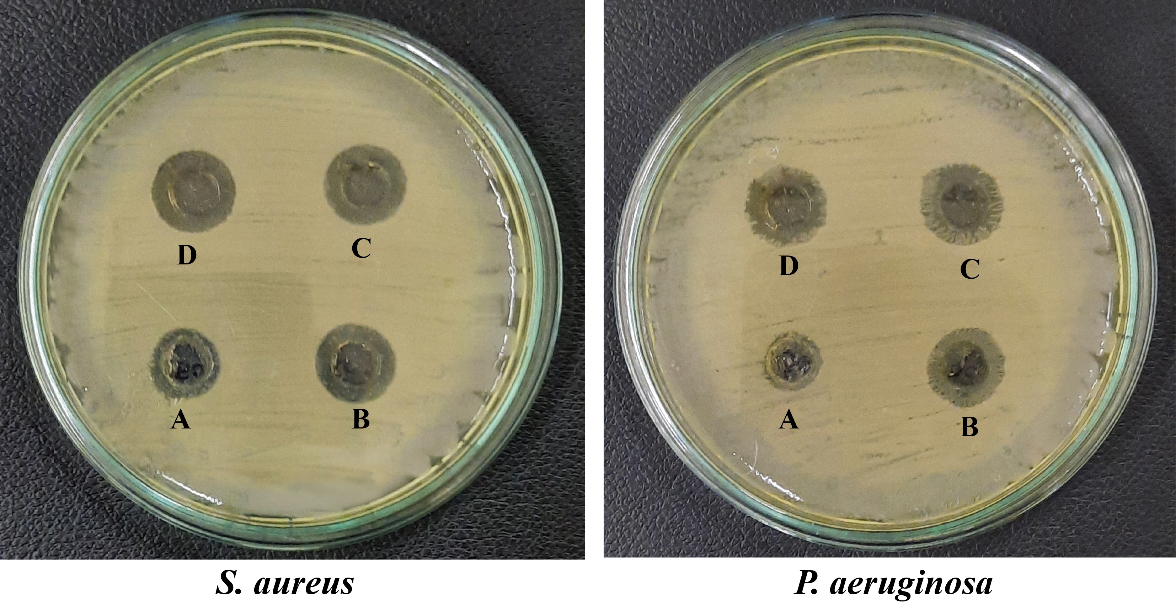
Figure S1.** Agar well diffusion assay of *S. aureus* and *P. aeruginosa* in the presence of the Ag-Cu Nps at concentrations of A-15, B-30, C-60, and D-120 μg/mL.

*S. aureus*

*P. aeruginosa*


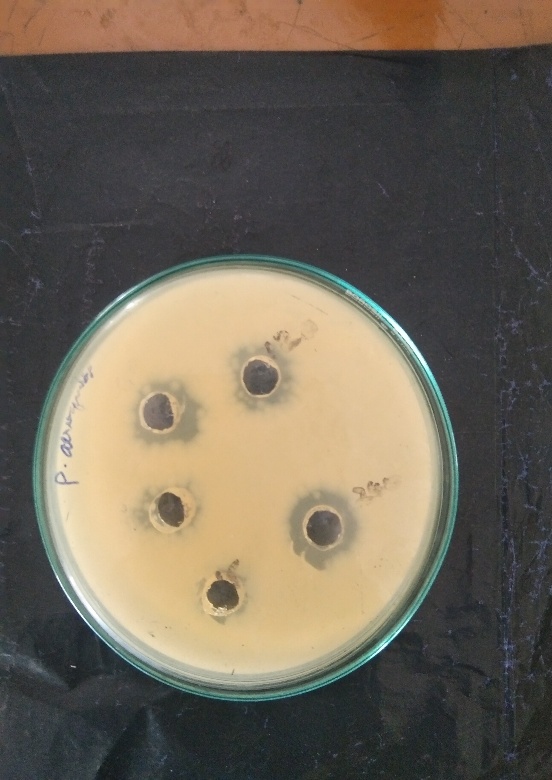


A

B

C

D

E


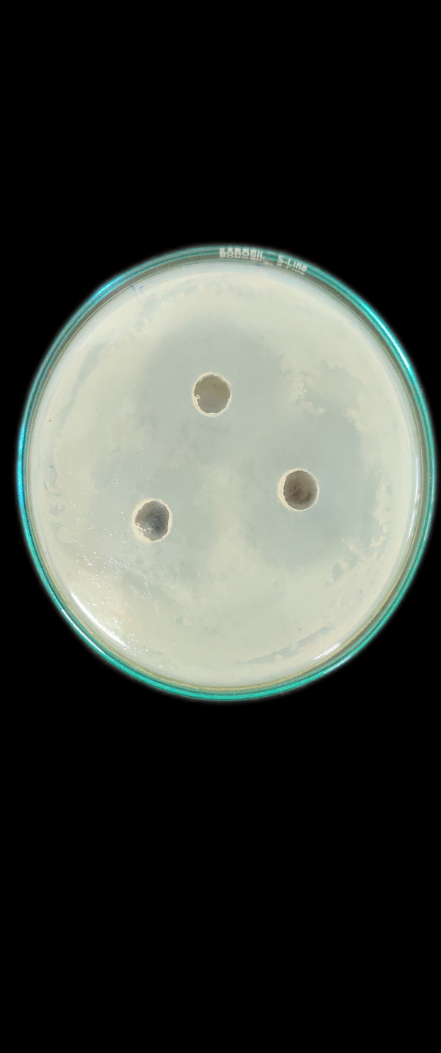


A

B

C


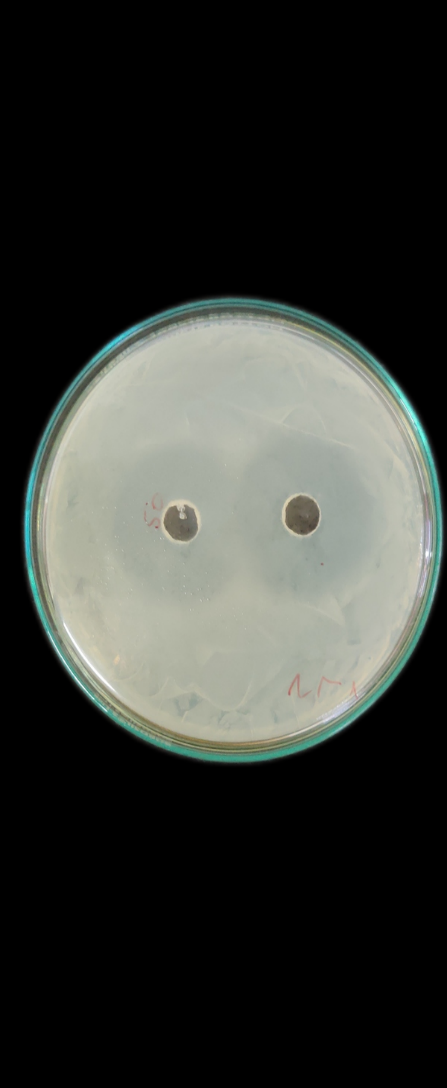


D

E

**Figure S2.** Agar well diffusion assay of *S. aureus* and *P. aeruginosa* in the presence of the antibiotic control Ampicillin at concentrations of A-15, B-30, C-60, D-120 and E-240 μg/mL.

120

15

30

240

**60**


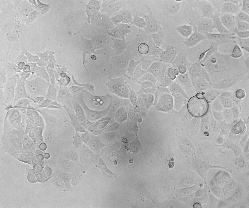

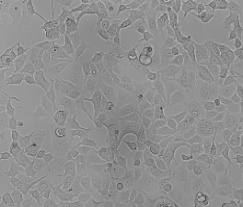

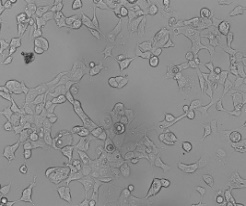

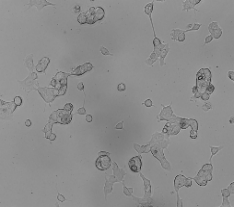

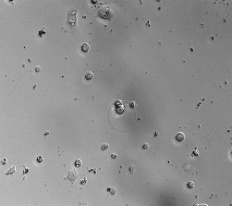

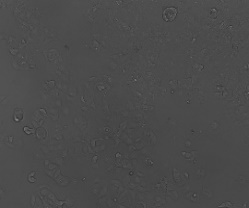

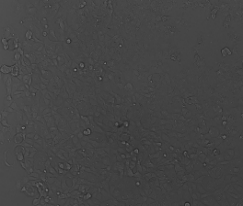

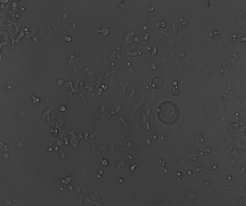

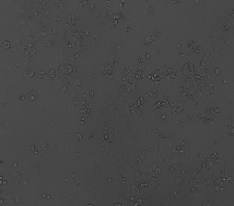

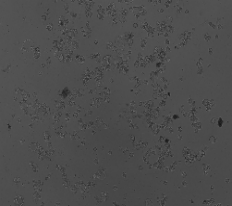

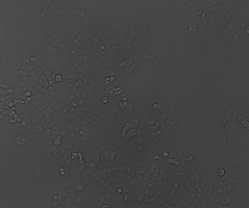

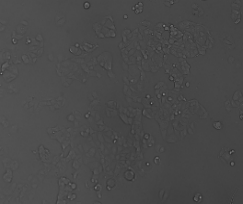

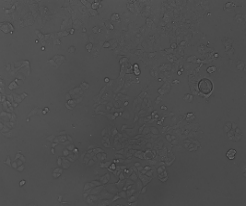

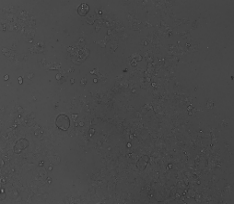

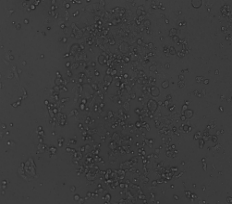


**(A)**

100

50

6.25

12.5

25

15

30

240

120

**(B)**

60

**(C)**

**Figure S3.** Microscopic examination of HEK293 cells following treatment with **(A)** Ag-Cu NPs (Conc. μg/mL), **(B)** Doxorubicin (Conc. μM/mL), and **(C)** *A. Lanata* Aq. Extract (Conc. μg/mL).


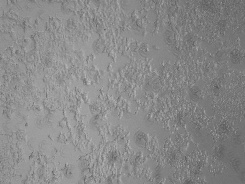

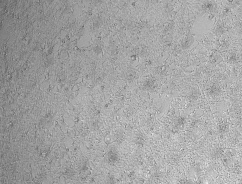

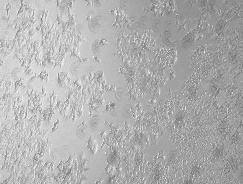

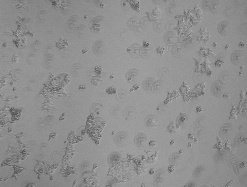

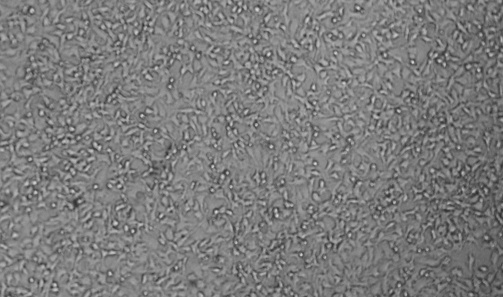

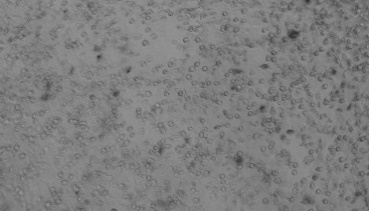

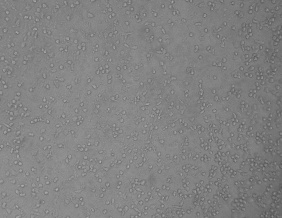

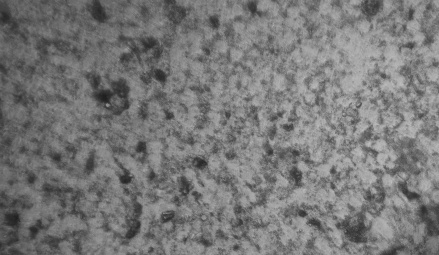

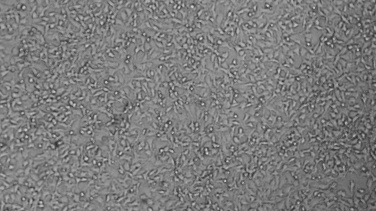

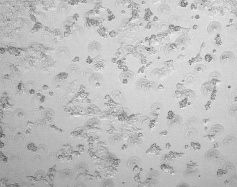

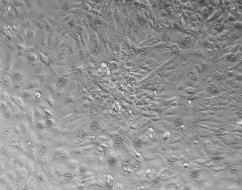

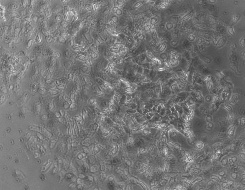

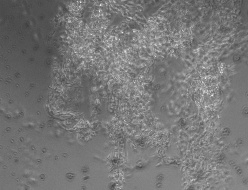

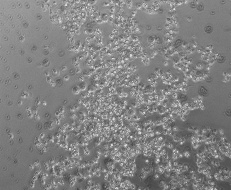

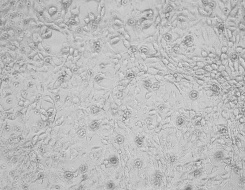


**25**

**50**

**6.25**

**12.5**

**100**

**60**

**120**

**15**

**30**

**240**

**60**

**120**

**15**

**30**

**240**

**(A)**

**(B)**

**(C)**

**Figure S4.** Microscopic examination of HeLa cells following treatment with **(A)** Ag-Cu NPs (Conc. μg/mL), **(B)** Doxorubicin (Conc. μM/mL), and **(C)** *A. Lanata* Aq. Extract (Conc. μg/mL).
